# Supplementary material for: A CNN-transformer fusion network for COVID-19 CXR image classification
Source: PLoS One. 2022 Oct 27;17(10):e0276758. doi: 10.1371/journal.pone.0276758 (PMC9612494; doi:10.1371/journal.pone.0276758)
Supplement: Attachment — Submitted filename: Response to Reviewers.docx [file pone.0276758.s001.docx]

Original Manuscript ID: PONE-D-22-24175

Original Article Title: “A CNN-transformer fusion network for COVID-19 CXR image classification”

To: PLOS ONE Editor

Re: Response to reviewers

Dear Editor,

Thank you for allowing a resubmission of our manuscript, with an opportunity to address the reviewers’ comments. We appreciate the efforts of you and reviewers who have contributed to this manuscript, whose work has helped improving quality of this paper. This manuscript has been revised according to the reviewers’ valuable advice.

We are uploading (a) our point-by-point response to the comments (below) (response to reviewers), (b) an updated manuscript with yellow highlighting indicating changes (Supplementary Material for Review), and (c) a clean updated manuscript without highlights (Main Manuscript).

Best regards,

Cao K et al.

Additional Editor Comments: The authors need to improve the description of methodology and compare the results with the state-of-the-art.

Author response: We gratefully appreciate your valuable suggestion. The description of the CNN architecture is added to the Feature focus sub-network in this manuscript. Meanwhile, the comparison with the Densenet121 model results is added. Besides, allow me to explain some reasons for comparing the results with the state-of-the-art. Table 4 compares the results of recent experiments by other research on Covid classification, for example, Civit-Masot 2020, Ozturk 2020, Apostolopoulos 2020, etc. Thank you again for your suggestion, and we will follow up the related new study in future.

Reviewer#1, Comment # 1: Discuss your proposition according to the newest approaches, especially in terms of other methods than a neural network. Why the proposed approach is worthy of investigation? For easier analysis, see such ideas as lung segmentation by a heuristic fox.

Author response: We gratefully appreciate your valuable suggestion. Lung segmentation by a heuristic fox for the extraction of lung features is very inspiring for our future work, and we will further verify the usefulness of the segmented features for our experiments. Some recent works regarding nature-inspired and heuristic optimization algorithms are cited in the part of Introduction.

Reviewer#1, Comment # 2: How did you model the architecture of CNN?

Author response: We gratefully appreciate your valuable suggestion. The description of the CNN architecture is added to the Feature focus sub-network in the manuscript.

Reviewer#1, Comment # 3: Some comparisons with learning transfer should be made.

Author response: We gratefully appreciate your valuable suggestion. We found some references on the classification of Covid-19 which uses transfer learning methods (Apostolopoulos, Ioannis D., and Tzani A. Mpesiana. "Covid-19: automatic detection from x-ray images utilizing transfer learning with convolutional neural networks." Physical and engineering sciences in medicine 43. 2 (2020): 635-640). This reference used the data from the Radiological Society of North America (RSNA), and conducted transfer learning of VGG19, MobileNet v2, Inception, Xception and Inception ResNet v2 with the accuracy of 93.48%, 92.85%, 92.85%, 92.85%, 92.85%. One of our datasets is also Radiological Society of North America (RSNA), and the fusion of local features and global features achieves better results than transfer learning method in the above reference, thus proving the effectiveness of our method.

Reviewer#1, Comment # 4: Discussion on results is very limited.

Author response: We gratefully appreciate your valuable suggestion. In the part of Discussion, different convolution kernel size is further discussed. In addition, the discussion regarding possible modules combinations is added. Our proposed network can extract different features and fuse features from different branches through mutual feature fusion to reduce the loss of useful feature information. Discussing the fusion of the local features with the global features, we conducted some experiments to test possible modules combinations, the results are shown in Table 5.

Reviewer#2, Comment # 1: "Covid-19 is a pulmonary disease caused by severe acute respiratory syndrome coronavirus 2 (SARS-CoV-2) in 2019 and is highly infectious, mutagenic, and Covid-19 and its novel mutant strains such as delta, omicron, and omicron XE variant have" - add the following two works for the support of this statement (A Healthcare System for COVID19 Classification Using Multi-Type Classical Features Selection; A Rapid Artificial Intelligence-based Computer-Aided Diagnosis System for COVID19 Classification from CT Images ).

Author response: We gratefully appreciate your valuable suggestion. Some new references have been added in the relevant places.

Reviewer#2, Comment # 2: Add the following studies in the related work, as the related work is not suffiecint and up to date.

- COVID19 Classification using Chest X-Ray Images: A Framework of CNN-LSTM and Improved Max Value Moth Flame Optimization

- COVID-19 Classification from Chest X-Ray Images: A Framework of Deep Explainable Artificial Intelligence.

Author response: We gratefully appreciate your valuable suggestion. The above two references are added in the part of Introduction.

Reviewer#2, Comment # 3: According to the WHO, as of April 4, 2022, there were 489,779,062 confirmed cases of COVID-19 including 6,152,095 deaths "- this statement need to be confirmed with correct data.

Author response: We gratefully appreciate your valuable suggestion. In the part of Introduction, we reexamined the cited report and update this statement as “According to the WHO weekly epidemiological update on COVID-19 (12 April 2022), as of 10 April 2022, over 496 million confirmed cases and over 6 million deaths have been reported globally”.

Reviewer#2, Comment # 4: There are a number of reasons for the lower sensitivity, such as the possible inadequate supply of reagents, the lack of expertise required for the testing, low viral load in patients and the long testing cycles."- this statement need some justifications.

Author response: We gratefully appreciate your valuable suggestion. Allow me to give some explanations for above comment. It is our uncorrected citation that caused this misunderstanding. The reasons for “the lower sensitivity, the possible inadequate supply of reagents, the lack of expertise required for the testing, low viral load in patients and the long testing cycles” are given in this report (Ref. [7]). We thank you for your correction, and have changed the position of the citation.

Reviewer#2, Comment # 5: "Medical imaging and deep learning (DL) can play an important role in pre-detection efforts to combat disease. In recent years, researchers have used deep neural networks to achieve remarkable results in a variety of fields. Recent advances in DL show that computers can extract more information from images, more reliably, and more accurately than ever before"- add the following works for the support of this important statement:

- Brain MRI analysis using deep neural network for medical of internet things applications

- BrainNet: optimal deep learning feature fusion for brain tumor classification

Author response: We gratefully appreciate your valuable suggestion. In the part of Introduction, some new references have been added in the relevant places.

Reviewer#2, Comment # 6: The major contributions should be revised, as the last contribution (3) is not a contribution.

Author response: We gratefully appreciate your valuable suggestion. We have deleted contribution (3) in the manuscript.

Reviewer#2, Comment # 7: Draw figure 1 in more attractive.

Author response: We gratefully appreciate your valuable suggestion. We have modified Figure 1 in this manuscript.

Reviewer#2, Comment # 8: It is important to add the images before and after the data augmentation.

Author response: We gratefully appreciate your valuable suggestion. The data augmentation image is shown in Figure 2 in Data augmentation.

Reviewer#2, Comment # 9: Remove Eq. 6-9, these are well known.

Author response: We gratefully appreciate your valuable suggestion. These equations have been removed.

Reviewer#2, Comment # 10: In table 7, what is reason behind the choice of these hyperparameters.

Author response: We gratefully appreciate your valuable suggestion. Table 2 shows the hyperparameters used in the experiments, in which we used scheduler = StepLR (optimizer, step_size=1, gamma=gamma), so that the learning rate would be adjusted at equal intervals for each epoch, and therefore a larger epoch and a widely used learning rate was chosen in order to find a better weight value.
